# Comparison of the long‐term outcomes of the self‐expandable metallic stent and transanal decompression tube for obstructive colorectal cancer

**DOI:** 10.1002/ags3.12235

**Published:** 2019-01-29

**Authors:** Ryuichiro Sato, Masaya Oikawa, Tetsuya Kakita, Takaho Okada, Atsushi Oyama, Tomoya Abe, Takashi Yazawa, Haruyuki Tsuchiya, Naoya Akazawa, Tetsuya Ohira, Yoshihiro Harada, Megumi Tanaka, Haruka Okano, Kei Ito, Takashi Tsuchiya

**Affiliations:** ^1^ Department of Gastroenterological Surgery Sendai City Medical Center Sendai Open Hospital Sendai Japan; ^2^ Department of Gastroenterology Sendai City Medical Center Sendai Open Hospital Sendai Japan

**Keywords:** obstructive colorectal cancer, retrospective study, self‐expandable metallic stent, survival rate, transanal decompression tube

## Abstract

**Aim:**

Endoscopic decompression using the self‐expandable metallic colonic stent (SEMS) or transanal decompression tube (TDT) can convert emergency surgery into elective one‐stage surgery for obstructive colorectal cancer (OCRC). The aim of the present study was to clarify the effect of SEMS and TDT on long‐term oncological outcomes.

**Methods:**

We retrospectively analyzed 76 consecutive pathological stage II and III OCRC patients who were inserted with SEMS or TDT as a bridge to curative surgery between 2009 and 2018.

**Results:**

There were 53 SEMS cases and 23 TDT cases. The tumor was located in the left colon in 58 cases and in the right colon in 18 cases. The interval between the decompression and the surgery was 16.5 days in the SEMS group and 13.0 days in the TDT group (*P = *0.09). Technical and clinical success rates were 100% and 100% for SEMS, and 95% and 91% for TDT, respectively. Stoma was created in four patients in the SEMS group, and in five in the TDT group (*P *=* *0.08). Three‐year overall survival rates of the SEMS and TDT groups were 82% and 86% (*P = *0.94), and disease‐free survival rates were 68% and 62% (*P = *0.79), respectively. The recurrence pattern was not significantly different.

**Conclusion:**

This study found no statistically significant differences between the effects of SEMS and TDT for OCRC as a bridge to surgery on long‐term outcomes.

## 1 INTRODUCTION

Intestinal obstruction is one of the common presenting symptoms of colorectal cancer. Its incidence is reported as high as 30%,^1^, ^2^ and obstructive colorectal cancer (OCRC) accounted for 85% of colonic emergency.^3^, ^4^ Emergency surgery is usually indicated and this is associated with increased morbidity, mortality, and stoma rate compared to elective surgery. Stoma creation is permanent in up to 40% of patients, and significantly diminishes patient's quality of life (QOL).^5^ Further, emergency surgery might result in oncologically suboptimal resection.^6^

With regard to right‐sided OCRC, resection with primary anastomosis is considered the treatment of choice.^7^ However, in some series, reported anastomotic leak rate was 2.5%~16.4%,^8^, ^9^ and mortality was higher compared to left‐sided OCRC,^9^, ^10^ suggesting choosing safer therapeutic options might be feasible in some cases. Management options for left‐sided OCRC, which accounts for 70% of OCRC,^6^, ^11^ are more diverse. The surgical strategies range from three‐stage surgery (proximal colostomy, tumor resection, and stoma closure) to one‐stage procedure. To avoid anastomotic leakage, Hartmann's procedure, subtotal colectomy with ileocolic anastomosis, and segmental resection followed by primary anastomosis with diverting stoma are occasionally selected based on surgeon's preference and patient's condition.^8^

Endoscopic decompression can convert emergency surgery into elective one‐stage surgery. Self‐expandable metallic colonic stent (SEMS) and transanal decompression tube (TDT) were both shown to be effective as a bridge to elective surgery, and associated with reduced morbidity and stoma rate compared to emergency surgery.^12^, ^13^, ^14^

Long‐term outcomes comparing SEMS and TDT have not been reported. The aim of the present study was to clarify the effect of SEMS and TDT on long‐term oncological outcomes.

## 2 METHODS

### 2.1 Patients

We retrospectively analyzed 76 consecutive pathological stage II and III OCRC patients who were inserted with SEMS or TDT as “a bridge to surgery” at Sendai City Medical Center between 2009 and 2018. All patients subsequently underwent curative surgical resection. Patients with benign disease, distant metastasis, positive surgical margin, and invasion from a non‐colonic malignancy were excluded from the study. There were 40 men and 36 women. Mean age of patients was 72.0 years (range, 37‐93), and median follow‐up time was 30.0 months (range, 0.6‐93). Postoperative complications were classified according to the Clavien‐Dindo classification.^15^

### 2.2 Diagnosis of OCRC

Diagnosis of OCRC was made by physical examination, contrast‐enhanced computed tomography (CT), contrast enema, and colonoscopy, and confirmed by histological examination. Pathological tumor staging was done according to the American Joint Committee on Cancer  (AJCC) cancer staging manual (7th edition).^16^ Colonic lesions proximal to the splenic flexure were defined as right‐sided tumors.

### 2.3 Endoscopic decompression

Decompression modality was chosen upon discussion between the surgeon and the endoscopist. For lower rectal cancer, we prefer using TDT to avoid SEMS migrating distally and interfering with transection of the distal rectum. Insertion of the SEMS or TDT was carried out by the endoscopist. A guidewire was introduced across the neoplastic stenosis under endoscopic and fluoroscopic guidance. For SEMS, Niti‐S colonic stent (TaeWoong Medical, Gimpo‐si, Korea) was deployed over the wire and through the scope without balloon dilatation. For TDT, the scope was removed leaving the guidewire in place, and Dennis Colorectal Tube (22‐Fr outer diameter and 145‐cm length; Coviden Japan, Tokyo, Japan) was inserted over the wire. The balloon at the tube tip was insufflated with 30 mL of water for fixation. The tube was flushed several times a day to prevent clogging. Technical success was defined as correct placement, and clinical success was defined as resolution of occlusive symptoms.

The colon proximal to the stenosis was evaluated by water‐soluble contrast enema, and colonoscopic examination was carried out after the surgery. Our institute introduced SEMS in 2013, and we have seen a moderate shift from TDT to SEMS thereafter.

### 2.4 Statistical analysis

Continuous variables are presented as mean ± SEM and were tested using the Mann‐Whitney *U*‐test. Associations between decompression modalities and clinicopathological parameters were evaluated in a cross‐table using the chi‐squared test. Overall survival (OS) and disease‐free survival (DFS) curves were generated according to the Kaplan‐Meier method, and were analyzed by the log‐rank test. StatView 5.0 software (SAS Institute Inc., Cary, NC, USA) was used for statistical analyses and differences with *P* values <0.05 were considered significant.

## 3 RESULTS

Clinicopathological findings of the 76 patients are summarized in Table 1. There were 53 SEMS cases, and 23 TDT cases. The tumor was located in the left colon in 58 cases, and in the right colon in 18 cases. TDT was advanced over the guidewire, and it was technically difficult to insert the tube beyond the hepatic flexure. There were four ascending colon cancer cases, in which only SEMS was placed mainly for this reason. Age, gender, tumor stage, and other clinicopathological parameters were comparable between the SEMS and the TDT groups, except there were more T3 tumors in the SEMS group (*P = *0.045).

**Table 1:** Association between decompression modalities and clinicopathological parameters in 76 colorectal cancer cases

Value	Decompression modality		*P*
	SEMS (n = 53)	TDT (n = 23)	
Age (y)^a^ [min‐max]	70.8 ± 1.7 [37‐90]	76.0 ± 2.4 [54‐93]	0.09
Gender			
Male	28	12	0.69
Female	25	11	
Tumor site			
Right	15	3	0.15
Left	38	20	
Ascending colon	4	0	0.48
Transverse colon	11	3	
Descending colon	11	4	
Sigmoid colon	20	11	
Rectum	7	5	
Stage			
II	24	10	0.88
III	29	13	
Depth of invasion (T stage)			
T3	40	12	**0.045**
T4	13	11	
Lymph node metastasis			
−	24	10	0.88
+	29	13	
Histological differentiation			
Well	27	14	0.43
Moderate + poor	26	9	
Vascular invasion			
−	19	10	0.44
+	34	12	
Lymphatic invasion			
−	5	3	0.59
+	48	19	

a Data are presented as mean ± SEM, and were evaluated by Mann‐Whitney *U*‐test. All other values represent the number of cases and were evaluated using a cross‐table using the chi‐squared test. *P*‐values <0.05 were considered significant, and are shown in boldface.

SEMS, self‐expandable metallic colonic stent; TDT, transanal decompression tube.

Interval between decompression and surgery was 16.5 days in the SEMS group and 13.0 days in the TDT group (*P = *0.09) (Table 2). Technical and clinical success rates were 100% and 100% for SEMS, and 95% and 91% for TDT, respectively. Drainage‐related complications were observed in one and two cases in the SEMS and TDT groups, respectively (*P = *0.16). A 74‐year‐old female patient with sigmoid colon cancer complained of mild abdominal pain after SEMS insertion. Perforation occurred during TDT placement for a 74‐year‐old male sigmoid colon cancer patient who underwent emergent sigmoidectomy with construction of a diverting stoma. His postoperative course was uneventful, and the stoma was reversed 2 months after the operation. However, he developed peritoneal dissemination 33 months after the first operation. Another patient required emergent Hartmann's operation 1 day after TDT placement as a result of inadequate drainage. This patient underwent a second operation on postoperative day 15 because she developed necrosis at the terminal ileum and in the remaining colon, resulting in perforation and localized peritonitis. This was the only case in the present study that showed obstructive colitis in the resected specimen.

**Table 2:** Perioperative data in 76 colorectal cancer cases

Value	Decompression modality		*P*
	SEMS (n = 53)	TDT (n = 23)	
Interval between drainage and operation (d)	16.5 ± 1.2 [5‐46]	13.0 ± 1.4 [0‐31]	0.09
Drainage‐related complications	1	2	0.16
Resumption of normal diet after drainage	32	0	**0.001**
Decrease in serum albumin (g/dl)^a^	0.57 ± 0.06	0.81 ± 0.09	**0.02**
Body weight loss (kg)^a^	1.6 ± 0.3	0.7 ± 0.5	0.55
Type of surgery			
Resection with primary anastomosis	49	18	0.08
Resection with diverting stoma	1	1	
Hartmann's procedure	3	4	
Laparoscopic resection (conversion)	11 (1)	2 (1)	0.20
Harvested lymph node			
<12	4	0	0.18
≧12	49	23	
Adjuvant chemotherapy			
−	26	13	0.55
+	27	10	
Postoperative hospital stay (d)	19.5 ± 1.6 [8‐77]	24.2 ± 4.5 [9‐102]	0.23

Change between decompression and surgery.

*P*‐values <0.05 were considered significant, and are shown in boldface.

SEMS, self‐expandable metallic colonic stent; TDT, transanal decompression tube.

Thirty‐two patients (60.4%) in the SEMS group were able to resume a normal diet after the drainage, and 10 patients were temporarily discharged and underwent preoperative evaluations on an outpatient basis, in contrast to none in the TDT group. Patients were given parenteral nutrition to meet nutritional requirements as needed. Decrease in serum albumin between decompression and surgery was significantly greater in the TDT group (*P *=* *0.02). Body weight loss during the interval was comparable between the groups (*P* = 0.55).

Forty‐nine patients (92%) in the SEMS group and 18 patients (78%) in the TDT group underwent curative resection with primary anastomosis. Stoma was created in four patients in the SEMS group including one diverting stoma, and five in the TDT group including one diverting stoma (*P = *0.08). Laparoscopic surgery was carried out in 11 cases in the SEMS group and in two cases in the TDT group. Conversion to open procedure was noted in one case in each group because of severe adhesion in one patient and obesity that resulted in a restricted operating field in another. At our institute, laparoscopic colectomy has been carried out for early‐stage patients, which explains the relatively low laparoscopic rate in this study. Currently, laparoscopic colectomy is indicated for all patients including endoscopically drained OCRC cases. Number of harvested lymph nodes was ≥12 in 49 (92%) and 23 (100%) cases in the SEMS and TDT groups, respectively (*P = *0.18). Adjuvant chemotherapy was given for 27 and 10 cases in the SEMS and TDT groups, respectively (*P = *0.55).

As shown in Table 3, postoperative complications were observed in 21 cases in the SEMS group and in seven cases in the TDT group (*P = *0.45). According to Clavien‐Dindo classification, most cases were grades I and II. Mortality rate was not significantly different, with one in the SEMS group (anastomotic leakage) and two in the TDT group (pneumonia; *P = *0.16). Postoperative hospital stay was 19.5 days in the SEMS group and 24.2 days in the TDT group (*P = *0.23).

**Table 3:** Postoperative complications in 76 colorectal cancer cases

Value	Decompression modality		*P*
	SEMS (n = 53)	TDT (n = 23)	
Postoperative complications	21	7	0.45
Ileus	8	2	
SSI	5	2	
Pneumonia	4	2	
Intestinal necrosis	0	1	
Anastomotic leakage	1	0	
Lymphorrhea	1	0	
Fever	1	0	
Diarrhea	1	0	
Grade I	10	2	0.12
Grade II	8	1	
Grade IIIa	2	0	
Grade IIIb	0	1	
Grade IV	0	1	
Grade V	1	2	

SEMS, self‐expandable metallic colonic stent; SSI, surgical site infection; TDT, transanal decompression tube.

**Figure 1:**
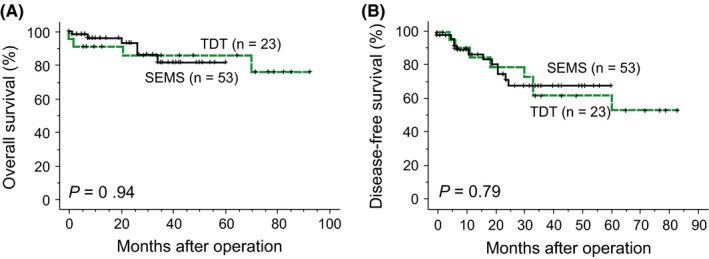
Overall survival and disease‐free survival curves of 76 pathological stages II and III colorectal cancer patients according to decompression modalities. A, Overall survival curves of all patients. B, Disease‐free survival curves of all patients. SEMS, self‐expandable metallic colonic stent; TDT, transanal decompression tube

**Figure 2:**
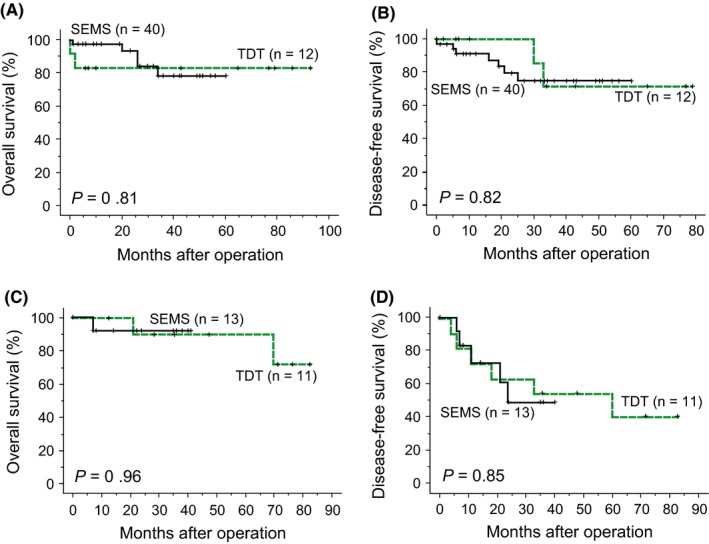
Overall survival (A) and disease‐free survival curves (B) of pT3 colorectal cancer patients. Overall survival (C) and disease‐free survival curves (D) of pT4 colorectal cancer patients. SEMS, self‐expandable metallic colonic stent; TDT, transanal decompression tube

**Figure 3:**
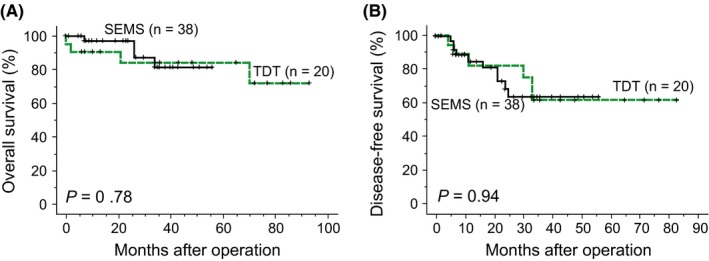
Overall survival (A) and disease‐free survival curves (B) of left‐sided colorectal cancer patients. SEMS, self‐expandable metallic colonic stent; TDT, transanal decompression tube

Three‐year OS rates in the SEMS and TDT groups were 82% and 86% (*P = *0.94), and DFS rates were 68% and 62% (*P = *0.79), respectively (Figure 1). When the cases were divided into T3 and T4, differences in OS and DFS were still non‐significant (Figure 2). When the cases were stratified by lymph node status, OS and DFS were not significantly different (*P *=* *0.67 and *P *=* *0.98 for lymph node‐negative cases, and *P *=* *0.78 and *P *=* *0.81 for lymph node‐positive cases, respectively). For left‐sided cases, OS and DFS rates of the SEMS and TDT groups were not significantly different (Figure 3). Survival analyses for right‐sided cases were not appropriate as the number of cases was small. There were 12 recurrent cases in the SEMS group and eight in the TDT group, and the recurrence pattern was not significantly different (*P = *0.17; Table 4).

**Table 4:** Sites of recurrent disease

Value	Decompression modality		*P*
	SEMS (n = 53)	TDT (n = 23)	
Liver	8	2	0.17
Lung	3	3	
Local recurrence	1	1	
Peritoneal dissemination	0	2	

SEMS, self‐expandable metallic colonic stent; TDT, transanal decompression tube.

## 4 DISCUSSION

Emergency surgery for OCRC is associated with increased morbidity and mortality compared to elective surgery.^5^ It is usually accompanied by multiple‐stage surgery with creation of temporary or permanent stoma which significantly compromises patient's QOL.^2^, ^5^, ^7^ Endoscopic decompression can convert emergency surgery into elective one‐stage surgery. Decompression allows bowel preparation, medical stabilization with correction of dehydration and electrolyte abnormalities, and optimization of comorbid illnesses, which theoretically result in lower morbidity, mortality, and stoma rate.

Self‐expandable metallic colonic stents were originally used for palliative intent for OCRC patients.^17^ Recently, they have been used as a bridge to surgery for potentially curable disease. However, concerns have been raised about the effect of colonic stents on short‐term complications and long‐term survival. Two randomized studies comparing SEMS and emergency surgery closed prematurely because of high perforation and technical failure rates in the stent group.^18^, ^19^ SEMS insertion was shown to increase cytokeratin 20 mRNA level in peripheral blood,^20^ and SEMS was associated with increased local recurrence.^1^ Studies including randomized controlled trials have provided conflicting results on long‐term outcomes of SEMS.^1^, ^18^, ^21^, ^22^ In the 2014 guidelines of the European Society of Gastrointestinal Endoscopy, SEMS placement as a bridge to elective surgery is not recommended as a standard treatment of symptomatic left‐sided malignant colonic obstruction.^23^

Transanal decompression tube is also used as a bridge to surgery, and it was associated with improved primary anastomosis rate and reduced morbidity compared with emergency surgery.^12^ Relative to SEMS, TDT is less costly,^24^ and it was popular in Japan, especially until 2012, because SEMS was not covered by national health insurance. TDT was comparable to SEMS in terms of postoperative morbidity and mortality,^24^, ^25^ but was associated with a higher permanent stoma rate.^26^ Unlike SEMS, TDT does not mechanically expand the tumor, and possibly has a smaller risk of tumor spread, which might eventually affect long‐term oncological outcomes. TDT might be more suitable therapeutic counterpart of SEMS than emergency surgery, since the therapeutic time course is similar. Only short‐term outcomes were available comparing the effect of SEMS and TDT as a bridge to surgery^12^, ^24^, ^25^, ^27^ and, to the best of our knowledge, this is the first study showing long‐term outcomes comparing these modalities.

Results of the present study showed that 3‐year OS and DFS were comparable between the SEMS group and the TDT group, and the recurrence pattern was not significantly different between the groups. Recent meta‐analyses showed that SEMS did not adversely affect long‐term results when compared with emergency surgery as a bridge to surgery,^4^, ^28^ and as palliative therapy.^29^ It was also reported that incidence of local and distant recurrence was not significantly different.^4^, ^28^ Our results were in line with these previous studies, suggesting that SEMS did not adversely affect long‐term oncological outcomes. TDT was equally effective in this regard, and could be another therapeutic option.

Studies on SEMS showed that perforation and subclinical silent perforation were associated with local recurrence and adverse long‐term outcomes.^18^, ^22^ Morbidity rate of SEMS differed among studies, and it was suggested that SEMS should be inserted by an experienced endoscopist.^21^ Reported perforation rate was 5.9%,^14^ which has been decreasing, and reaching 0% in some studies^24^, ^30^ including ours. In the present study, we experienced one perforation case in the TDT group who developed peritoneal dissemination 33 months after the operation. Postoperative complication rate was comparable between the groups in this study. Matsuda et al^24^ reported that SEMS was associated with reduced incidence of surgical site infection (SSI) compared to TDT. They attributed this to the high proportion of laparoscopic surgery in the SEMS group, which might explain the similar SSI rate observed in our study.

In the present study, endoscopic decompression was applied not only for left‐sided OCRC but also for right‐sided cases. As for right‐sided OCRC, emergency colectomy with primary anastomosis is the standard of care in Western countries.^7^ However, emergency colectomy for right‐sided OCRC was associated with increased morbidity and mortality,^7^, ^9^, ^10^, ^26^ and it is a challenge for surgeons and anesthesiologists to manage the patient in a suboptimal condition in the emergency setting. In a retrospective study of 776 patients in France, postoperative morbidity and mortality rates for emergency surgery for right‐sided OCRC were 51% and 10%, respectively, and age >70 years, American Society of Anesthesiology (ASA) score ≥3, and hemodynamic instability at admission were independent predictors for postoperative mortality.^9^ Repici et al^31^ reported that placement of SEMS into the proximal colon was safe and effective. The study comparing SEMS and TDT for right‐sided OCRC demonstrated similar technical success and morbidity rates, but clinical success rate was significantly higher in the SEMS group owing to higher decompression efficacy.^32^ Kye et al^3^ reported that postoperative morbidity, 5‐year OS and DFS were comparable between the SEMS group and the emergency surgery group for right‐sided OCRC. In the present study, although the number was small (n = 15), 3‐year OS and DFS of SEMS‐inserted right‐sided OCRC cases were 100% and 82%, respectively. SEMS might be feasible for some right‐sided OCRC patients, especially for those at high surgical risk as a bridge to surgery and as palliative therapy, but this awaits further study.

Self‐expandable metallic colonic stents might have some nutritional advantage over TDT, and patients in the SEMS group might have better QOL than those in the TDT group. SEMS restored luminal patency which resulted in better drainage efficacy.^24^, ^27^, ^32^ More than half of the patients resumed a normal diet in this study and there were 10 patients in the SEMS group who were discharged and received preoperative evaluations on an outpatient basis. TDT is relatively narrow and patients were normally allowed only liquid. Although all patients in this study were properly managed to meet nutritional requirements, decrease in serum albumin between decompression and surgery was significantly greater in the TDT group. TDT is usually removed at surgery and patients had to bear the discomfort of the tube until surgery. TDT was associated with clogging which required irrigation several times a day. The advantages of SEMS might counterbalance its high cost, but further studies are warranted to evaluate the cost‐effectiveness, and QOL.

This study was limited by the small sample size, and its retrospective, non‐randomized design in a single institution. Although we limited patients to pathological stages II and III cases, heterogeneity existed in patients’ backgrounds. Moreover, median follow‐up time was relatively short, and there was a systematic difference in observation period between the SEMS and TDT groups (26.0 and 43.0 months as median values, respectively). The results therefore must be interpreted with caution.

In summary, the present study found no statistically significant differences between the effects of SEMS and TDT for OCRC as a bridge to surgery on long‐term outcomes. Future research with a large sample size and a longer observation period is warranted to confirm the present findings. Considering QOL, the wider applicability including for the right colon, and the global popularity of SEMS make it a possibly better option, but the indications for this treatment need to be clarified. SEMS should be used in institutions with expertise in endoluminal stenting to minimize complications.

## DISCLOSURE

Conflicts of Interest: Authors declare no conflicts of interest for this article.

Ethical Statement: The protocol for this research project was approved by the Ethics Committee of the institution (#2018‐0027) and it conforms to the provisions of the Declaration of Helsinki.
